# Beta-Adrenergic Blockade in Advanced Non-Small Cell Lung Cancer Patients Receiving Immunotherapy: A Multicentric Study

**DOI:** 10.7759/cureus.52194

**Published:** 2024-01-13

**Authors:** Ana Duarte Mendes, Ana Rita Freitas, Rodrigo Vicente, Ricardo Ferreira, Telma Martins, Maria João Ramos, Carlota Baptista, Bruno Miguel Silva, Inês Margarido, Marina Vitorino, Michelle Silva, Sofia Braga

**Affiliations:** 1 Medical Oncology Department, Hospital Professor Doutor Fernando Fonseca, Amadora, PRT; 2 Medical Oncology Department, Centro Hospitalar Universitário de Santo António, Porto, PRT; 3 Medical Oncology Department, Hospital Beatriz Ângelo, Loures, PRT; 4 Medical Oncology Department, Hospital da Luz Lisboa, Lisboa, PRT

**Keywords:** immunotherapy, beta-adrenergic signaling, immune response, immune checkpoint inhibitor, lung cancer

## Abstract

Introduction

The standard treatment of cancer has dramatically improved with immune checkpoint inhibitors (ICIs). Despite their proven advantage, many patients fail to exhibit a meaningful and lasting response. The beta-adrenergic signalling pathway may hold significant promise due to its role in promoting an immunosuppressive milieu within the tumour microenvironment. Inhibiting β-adrenergic signalling could enhance ICI activity; however, blocking this pathway for this purpose has yielded conflicting results. The primary objective of this study was to evaluate the effect of beta-blocker use on overall survival and progression-free survival during ICI therapy.

Methods

A multicentric, retrospective, observational study was conducted in four Portuguese institutions. Patients with advanced non-small cell lung cancer treated with ICIs between January 2018 and December 2019 were included. Those using beta blockers for non-oncological reasons were compared with non-users.

Results

Among the 171 patients included, 36 concomitantly received beta blockers and ICIs. No significant increase was found in progression-free survival among patients who took β-blockers (HR 0.74, 95% confidence interval (CI) 0.48-1.12, p = 0.151), and no statistically significant difference was found in overall survival. An apparent trend was observed towards better outcomes in the beta-blocker group, with a median overall survival of 9.93 months in the group not taking β-blockers versus 14.90 months in the β-blocker group (p = 0.291) and a median progression-free survival of 5.37 in the group not taking β-blockers versus 10.87 months in the β-blocker group (p = 0.151). Nine (25%) patients in the beta-blocker group and 16 (12%) in the non-beta-blocker group were progressive disease-free at the end of follow-up. This difference between the two groups is statistically significant (p = 0.047).

Conclusion

Our study found no statistically significant evidence that beta blockers enhance the effectiveness of immunotherapy. Using adrenergic blockade to modulate the immune system shows promise, warranting the need to develop prospective clinical studies.

## Introduction

Lung cancer is the second most common cancer and the leading cause of cancer-related death globally [1]. Although there have been significant improvements in diagnosis and treatment, the long-term survival rate of patients with non-small cell lung cancer (NSCLC), especially metastatic cancer, remains poor [2]. Nevertheless, there has been a gradual improvement in the overall survival rates for these patients over the past decade. The 5-year survival rate for all stages combined is 28% [3].

Stress response has been recognized as contributing to tumorigenesis and cancer progression [4]. Stress has been found to promote the development of lung cancer in animal models of carcinogen-induced lung cancer [5,6]. Stress leads to increased levels of catecholamines, such as norepinephrine and epinephrine, which have long been believed to affect overall health [7,8]. Adrenergic receptors mediate the biological effects of stress hormones and are categorized into two subtypes, α and β. β-adrenergic receptors (βARs) are expressed in normal tissue and overexpressed in various cancers including colon, lung, and breast cancer [9,10]. In the tumor microenvironment, cancer and immune cells often express βARs [11]. The βAR pathway regulates the fight-or-flight stress responses driven by the sympathetic nervous system [12]. These pathways mediate cellular responses to various stimuli, including stress and inflammation [13]. When epinephrine or norepinephrine binds to these receptors, it triggers the synthesis of cyclic adenosine monophosphate (AMP) by adenylyl cyclase, which then activates protein kinase A. Activating βARs can also stimulate other signal transduction pathways, including the mitogen-activated protein kinase/extracellular signal-regulated kinase pathway [14]. These pathways regulate cells’ survival and growth, activating transcription factors such as NF-kB and the CREB family. Transcription regulators are responsible for controlling the expression of several genes, including interleukin-8, interleukin-6, matrix metalloproteinases, and vascular endothelial growth factor. These genes can promote inflammation, cellular invasion, and angiogenesis [6,15-17]. Adrenergic signaling significantly hinders T-cell activation, differentiation, and function and is intrinsically associated with immune function [6]. Although the precise effect of stress on immunity remains unclear, animal studies have indicated that minimizing stress can considerably boost the immune response to tumours and enhance the efficacy of conventional cancer therapies. Conversely, stress-triggered βAR signalling may impede the immune system, resulting in a surge of immune suppressive cells, a decline in cytokine expression that encourages T-cell development, and a decrease in T-cell cytotoxicity [18-21]. Chronic adrenergic signalling suppresses effector CD8+ T cells in the tumour microenvironment in animal models [17,22].

In their recent study, Globig et al. describe a link between stress catecholamines and the progression of T-cell exhaustion through the β1 adrenergic receptor. CD8+T cells play a critical role in the anti-tumour immune response and can undergo a state designated by exhaustion. In this state, the CD8+ cells increase their expression of β1 adrenergic receptor, and the consequent exposure to catecholamines suppresses cytokine production and cell proliferation [23].

Immunotherapy, such as immune checkpoint inhibitors (ICIs), has revolutionized lung cancer treatment by boosting anti-cancer immune responses. Despite the proven clinical advantage, however, some tumours do not respond to ICIs, highlighting the need for predictive biomarkers of response [24,25]. ICI outcomes have been linked to various factors, including tumour mutational burden, hypoxia, interferon-γ, the microbiome, extracellular matrix, molecular and cellular characterization within the tumour microenvironment, and programmed death-ligand 1 (PD-L1) expression [24,26]. The use of PD-L1 as a biomarker has been a subject of debate, largely attributable to its heterogeneous and dynamic nature. The incomparability of results among trials could result from using different immunohistochemistry platforms, different cutoff values, and scoring systems. Additionally, the variability in PD-L1 expression, which could be due to various cellular mechanisms such as genomic aberrations, control mechanisms for transcription and translation, RNA/protein stability, and host-microbiome immunoediting, can lead to a misinterpretation of the actual status of PD-L1 [27,28].

Several strategies are currently under investigation to improve ICI response. Recently, strategies targeting the tumour microenvironment have been developed to achieve robust immunotherapeutic responses. For example, recent developments in biomedical engineering and nanotechnology show promising possibilities for delivering immunoregulatory agents [29-31]. Among the most promising avenues of inquiry are indirect immune modulation and direct enhancement of tumour cytotoxicity [32-34]. There has been a growing interest in beta-adrenergic blockade as a contributor to tumorigenesis, tumour progression, and metastasis [35,36]. βAR signalling can be inhibited pharmacologically with antagonists. βAR antagonists represent a widely prescribed medication for managing various medical conditions, including but not limited to heart failure, essential tremor, hypertension, acute myocardial infarction, anxiety disorders, migraines, and glaucoma [37,38]. Beta-blockers are commonly used in medical practice and can be classified into two primary categories: pan-β-blockers and β1-selective blockers. The former targets both β1ARs and β2ARs, while the latter solely targets β1Ars [21]. Existing research has demonstrated a positive correlation between the use of beta blockers for non-cancer-related conditions and cancer outcomes [39].

Furthermore, as described in preclinical studies, beta-blocker treatment could convert tumours to an immunologically active phenotype [40,41]. Combination therapy with βAR blockade could be an appealing strategy to modulate the immune system.

The primary objective of this study was to assess the effect of beta-blocker use on overall survival (OS) and progression-free survival (PFS) during ICI therapy.

## Materials and methods

Research approach

This study was conducted retrospectively across four oncological centres in Portugal (Hospital Professor Doutor Fernando Fonseca, Centro Hospitalar Universitário de Santo António, Hospital Beatriz Ângelo, and Hospital da Luz Lisboa) and involved 171 patients.

Participants

All patients were >18 years of age. Eligibility criteria included patients with histologically or cytologically confirmed non-small cell lung cancer, stage IV (American Joint Committee on Cancer, 8th edition), who were treated with ICIs either in monotherapy or in combination with chemotherapy from January 2018 to December 2019. Exclusion criteria included patients who discontinued beta blockers for any reason within three months of starting ICIs.

Data collection

The medical reports were reviewed to collect data regarding beta-blocker use, defined as using any beta blocker used for non-oncological reasons when initiating ICI treatment. We gathered comprehensive information including demographic data, such as age and gender, as well as performance status and smoking status. Tumour characteristics such as histology, PD-L1 status, and disease burden, as well as survival outcomes were also retrieved.

Research ethics

This study was conducted in compliance with the 2013 revision of the Helsinki Declaration. The study’s approval was granted by the Ethical Committee for Health of Hospital Professor Doutor Fernando Fonseca (approval No. 101/2023).

Data analysis

For continuous variables, descriptive results were expressed, depending on the normality of their distribution, as mean and standard deviation or as median and interquartile range. For categorical data, variables were tested for correlation using Pearson’s chi-squared test. For numerical data, the Mann-Whitney U-test or the independent samples t-test were used, depending on the normality of their distribution. The defined outcomes of our study are survival outcomes, with OS and PFS as outcome measures, as follows: OS was defined as the interval between treatment initiation and the event of mortality, regardless of any underlying cause. PFS was defined as the interval between treatment initiation and the first occurrence of either disease progression or death. Kaplan-Meier analysis was used to determine the difference in survival outcomes between the beta blocker and the non-beta blocker groups. The level of significance to reject the null hypothesis was set at an α level ≤ 0.05. We employed both univariate and multivariate Cox regression models to examine the potential association between clinical variables and the outcome variable, PFS. The explanatory variables, apart from beta-blocker use, were defined a priori based on data from the literature. The statistical analysis was performed using SPSS version 22.0 (IBM Corporation, Armonk, NY, USA).

## Results

A total of 171 patients were identified, and 36 (21.1%) were on beta blockers. The main reason for beta-blocker use in our population was heart failure (36%) and hypertension (25%). The baseline characteristics of the population are shown in Table 1.

**Table 1:** Baseline Characteristics CNS = Central Nervous System; ECOG PS = Eastern Cooperative Oncology Group Performance Status; PD-L1 = Programmed Death-ligand 1; SD = Standard Deviation.

Characteristic	No beta blocker (n=135)	Beta blocker (n=36)	p-value
Age (years), mean ± SD	62.9±9.4	65.8±8.9	0.092
Sex, n (%)			0.523
Male	99 (73.3)	26 (72.2)	
Female	36 (26.7)	10 (27.8)	
ECOG PS, n (%)			0.503
0	42 (31.1)	9 (25.0)	
1	80 (59.3)	25 (69.4)	
2	13 (9.6)	2 (5.6)	
Smoking Status, n (%)			0.573
Current or former	109 (80.7)	29 (80.6)	
Never	26 (19.3)	7 (19.4)	
Weight Loss at Diagnosis, n (%)			0.120
Yes	45 (33.3)	11 (30.6)	
No	90 (66.7)	25 (69.4)	
Histology, n (%)			0.306
Adenocarcinoma	99 (73.3)	21 (58.3)	
Squamous cell	32 (23.7)	13 (36.1)	
Other	4 (3.0)	2 (5.6)	
Tumour PD-L1 Status, n (%)			0.970
<1%	41 (30.4)	10 (27.8)	
1–49%	38 (28.1)	10 (27.8)	
>50%	55 (40.7)	15 (41.7)	
Unknown	1 (0.8)	1 (2.7)	
CNS Disease, n (%)			0.627
Yes	26 (19.3)	5 (13.9)	
No	109 (80.7)	31 (86.1)	
Line of Treatment, n (%)			0.906
First	58 (43.0)	14 (38.9)	
Second	59 (43.7)	17 (47.2)	
Third or higher	18 (13.3)	5 (13.9)	
Treatment, n (%)			0.098
Pembrolizumab	81 (60.0)	16 (44.4)	
Nivolumab	50 (37.0)	17 (47.3)	
Atezolizumab	4 (3.0)	3 (8.3)	
Concurrent Chemotherapy, n (%)			0.183
Yes	13 (9.7)	1 (2.7)	
No	122 (90.3)	35 (97.3)	

The two groups in the study were similar in terms of age, sex, performance status, smoking status, weight loss history, and histology. In addition, almost all patients (98.8%) had information available regarding their tumour PD-L1 status, and there were no differences observed between the two groups in terms of PD-L1 expression. Furthermore, there were no differences between the groups in terms of the line of treatment and the type of ICI used. Moreover, most patients who took beta blockers used selective beta blockers (28 out of 36, 77.8%). Patients who were taking beta blockers had a median follow-up time of 23.11 months, and those who were not had a median follow-up time of 18.30 months.

**Figure 1:**
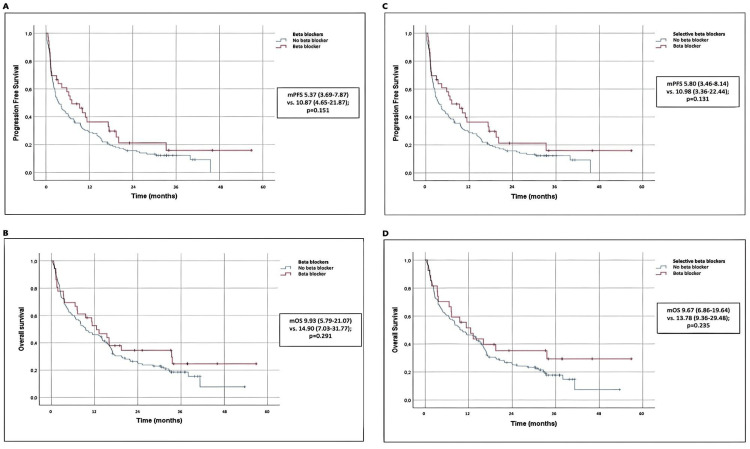
Survival Outcomes (A) Concomitant use of beta blockers in NSCLC patients under immunotherapy was not associated with significant prolonged progression-free survival or overall survival. (B) The analysis of the subset of patients with selective beta blockers also did not reveal a longer PFS (C) or OS (D).

Our study found no significant improvement in PFS among the group of patients who took beta blockers compared to those who did not. The beta blocker group had a median PFS of 10.87 months, while the non-beta blocker group had a median PFS of 5.37 months. This difference was not statistically significant (p = 0.151) (Figure 1A). Similarly, there was no significant difference in median OS between the two groups, with the beta blocker group having a median OS of 14.90 months and the non-beta blocker group having a median OS of 9.93 months (p = 0.291) (Figure 1B). The results did indicate some clinical benefit, however, as the beta blocker group had a doubling of median PFS and a clinically meaningful increase in median OS. A subset analysis of patients who were taking selective beta blockers also did not reveal an extended progression-free survival or overall survival (Figure 1C-1D).

Notably, nine (25%) patients in the beta-blocker group and 16 (12%) patients in the non-beta-blocker group were progressive disease-free at the end of follow-up, a difference that is statistically significant between the two groups (p = 0.047) (Table 2).

**Table 2:** Efficacy Outcomes CI = Confidence Interval.

Variable	No beta blocker (n=135)	Beta blocker (n=36)	p-value
Progressive Disease, n (%)	119 (88)	27 (75)	0.047
Deaths, n (%)	108 (80)	25 (69)	0.176
Median Progression-Free Survival, in months (95% CI)	5.37 (3.69–7.87)	10.87 (4.65–21.87)	0.151
Median Overall Survival, in months (95% CI)	9.93 (5.79–21.07)	14.90 (7.03–31.77)	0.291

We conducted a comprehensive analysis of several clinical variables, such as age, sex, and tumour PD-L1 status, using a uni- and multivariate Cox proportional hazards regression model (Table 3). Our primary goal was to determine the possible association between these variables and PFS. Upon analysing the data, we observed that beta-blocker use did not result in any statistically significant effect on PFS. However, we found a strong correlation between tumour PD-L1 positivity (p = 0.014) and a prolonged PFS.

**Table 3:** Multiple Linear Regression Associations of Progression-Free Survival CI = Confidence Interval; CNS = Central Nervous System; ECOG PS = Eastern Cooperative Oncology Group Performance Status; HR = Hazard Ratio; PD-L1 = Programmed Death-Ligand 1.

	Univariate Cox			Multivariate Cox		
Clinical variable	HR	95% CI	p-value	HR	95% CI	p-value
Beta Blockers (Yes vs. No)	0.74	0.48–1.12	0.151	0.78	0.50–1.20	0.250
Age	1.00	0.98–1.02	0.961	1.00	0.98–1.02	0.770
Sex (Male vs. Female)	0.91	0.63–1.32	0.623	1.07	0.70–1.63	0.765
Weight Loss (Yes vs. No)	1.40	0.99–1.98	0.054	1.31	0.89–1.93	0.177
Smoking (Yes vs. No)	0.97	0.64–1.47	0.895	1.11	0.68–1.79	0.682
ECOG PS (0/1 vs. 2)	1.42	0.80–2.51	0.233	1.55	0.84–2.84	0.157
CNS Disease (Yes vs. No)	0.77	0.50–1.19	0.240	0.85	0.53–1.37	0.504
PD-L1 Status (Positive vs. Negative)	0.56	0.39–0.80	0.002	0.59	0.38–0.90	0.014
Line of Treatment (1^st^/2^nd^ vs. Higher)	1.28	0.92–1.78	0.144	1.14	0.76–1.70	0.528
Concurrent Chemotherapy (Yes vs. No)	0.76	0.42–1.38	0.371	0.762	0.41–1.43	0.398

## Discussion

We sought to determine the influence of beta-blocker use on survival outcomes of metastatic NSCLC patients treated with immunotherapy.

Our retrospective study found no statistically significant correlation between the use of beta blockers and improvement in PFS or OS. A trend towards better outcomes was found, however, with an improvement in median PFS and median OS. In the linear regression analysis, PD-L1 positivity was significantly associated with prolonged PFS. This result is consistent with the available literature, given that PD-L1 expression is widely accepted as a predictor of response.

In our study, both the beta blocker and non-beta blocker groups were found to be evenly matched in terms of their respective demographics. The statistical analysis of the groups revealed no statistically significant difference between them. A few differences between the two groups could skew our results, however. The patients in the beta-blocker group were older, which may be explained by the fact that the main reasons for beta-blocker treatment are cardiovascular comorbidities whose incidence increases with age [42]. This group also had fewer patients with an ECOG PS of 0 (25% vs. 31%). The incidence of squamous carcinoma was also higher (36% vs. 24%) in the beta blocker group. Moreover, the percentage of patients in the beta-blocker group who were receiving ICIs as monotherapy was also higher (97% vs. 90%). However, the rate of receiving ICIs as first-line therapy was lower in the beta-blocker group (39% vs. 43%), which may have led to biased results favouring the non-beta-blocker group. Numerous analyses have illustrated that PFS is notably longer when ICIs are implemented as the first line of metastatic NSCLC as opposed to its use in the second line setting [43,44].

Conversely, patients taking beta blockers had a lower incidence of central nervous system disease (14% vs. 19%), which is a poor prognosis factor that could bias the results in favour of the beta-blocker group [45]. We also examined the effect of selective beta blockers but found no significant differences in survival outcomes [46]. A more substantial effect with non-selective beta blockers has been described, but the recent data by Globig et al. could open the door to selectively blocking the β1 adrenergic receptor [23].

Retrospective data regarding the effect of beta blockers on the outcomes of ICI in NSCLC is sparse and contradictory. To our knowledge, Oh et al. were among the few authors to investigate the possible effect of beta blockers on immunotherapy outcomes in patients with metastatic lung cancer. They describe a positive association concerning the effect of beta blocker on PFS (HR 0.58, 95% CI 0.36-0.93) [47]. A meta-analysis by Yan et al. sought to investigate the prognostic effect of beta-blocker administration in solid cancer patients undergoing immunotherapy. They found that beta-blocker use had no significant effect on the OS or PFS of the patients. However, beta blockers were significantly associated with a superior objective response to ICI, with an odds ratio of 0.42 (0.19-0.94) and a p-value of 0.036. Additionally, in the subgroup of patients with lung cancer, the odds ratio was 0.25 (0.08-0.83) with a p-value of 0.024 [48].

Despite the lack of statistical significance in our results, a few points should be highlighted. First, to our knowledge, little data has been published regarding the possible association between beta blockers and immunotherapy in lung cancer, and our study is the first with a multicentre design. Second, we did find a trend towards better outcomes, the relevance of which should be analysed with caution, as statistical significance was not reached. We also found a statistically significant difference in the progressive disease-free rate at the end of the follow-up: 25% of patients in the beta-blocker group and 12% of patients in the non-beta-blocker group were progressive disease-free (p = 0.047). Previous studies suggest that adrenergic blockade has an effect on outcomes, but the exact mechanism has yet to be defined and therefore cannot yet be targeted. In addition to the precise mechanism, other questions arise. Could the chronicity of the therapy alter βAR expression and sensitivity? Are there βAR resistance mechanisms regarding beta blockers in the tumour microenvironment? If we were to use beta blockers as adjunctives to oncological treatments purposefully, what would be the best timing for beta blocker initiation, and what would be the most suitable disease setting? Some randomized clinical trials are currently testing the tolerability and efficacy of adding a beta blocker to immunotherapy in solid cancers, namely adding propranolol to standard immunotherapy in angiosarcoma or melanoma [49,50].

Our study’s retrospective design and small sample impose limitations on the interpretation of the findings. Additionally, the substantial percentage of patients who were administered ICIs as a subsequent line of treatment, as opposed to complying with the current guidelines, further accentuates these limitations. Any conclusions drawn from the study should take into account these limitations, and further research should be conducted to validate the results.

## Conclusions

We sought to determine the effect of βAR blockade on survival outcomes in NSCLC patients treated with immunotherapy.

Our study found no association between beta-blocker use and improved PFS or OS. However, a trend was observed towards better outcomes, and the theoretical potential of adrenergic blockade in modulating the immune system seems appealing. Further studies are warranted to explore adrenergic blockade's full potential.
